# Severe Metabolic Acidemia in a Patient with Aleukemic Leukemia

**DOI:** 10.1155/2018/1019034

**Published:** 2018-11-18

**Authors:** Moutaz Ghrewati, Faiza Manji, Varun Modi, Chandra Chandran, Michael Maroules

**Affiliations:** ^1^Resident PGY-2 in the Internal Medicine Department at St. Joseph's University Medical Center, 703 Main St, Paterson, NJ 07503, USA; ^2^Fellow PGY-6 in the Hematology Oncology Department at St. Joseph's University Medical Center, 703 Main St, Paterson, NJ 07503, USA; ^3^Program Director of Internal Medicine Residency at St. Joseph's University Medical Center, 703 Main St, Paterson, NJ 07503, USA; ^4^Program Director of Hematology Oncology Fellowship at St. Joseph's University Medical Center, 703 Main St, Paterson, NJ 07503, USA

## Abstract

Malignancy associated lactic acidosis is a rare metabolic complication that may accompany various types of malignancies. To date, most cases that have been reported are associated with hematologic malignancies (lymphoma and leukemia). Many theories have been proposed to explain the pathophysiology of lactic acidosis in malignancies. We are reporting an unusual case of a 62-year-old female who presented with a complaint of generalized weakness. Patient was found to have pancytopenia and metabolic acidosis with an anion gap secondary to lactic acid in addition to non-anion gap acidosis (NAGA). The lactic acidosis resolved only after initiation of chemotherapy as she was diagnosed with B-cell acute lymphoblastic leukemia. Our patient also had a coexistent Renal Tubular Acidosis (RTA) with large kidneys. The kidney size also decreased with chemotherapy. Our case is unique as evidenced by aleukemic leukemia combined with anion gap acidosis and non-anion gap acidosis. Lactic acidosis has many different causes; although rare, hematologic malignancies should be included in the differential diagnosis regardless of cell counts or tumor burden.

## 1. Introduction

Lactic acidosis is classified based on tissue perfusion and oxygenation into type A and type B. Type A occurs when there is marked decrease in oxygen delivery to tissues. On the other hand, type B lactic acidosis occurs in the presence of sufficient oxygen delivery to tissues with main causes being malignancy, diabetes mellitus, drugs, hepatic failure, and renal failure [1].

Lactic acidosis has been reported in many cases of leukemia as being associated with an elevated white blood cell count. However, lactic acidosis can still occur even when leukemia is present with a low white blood cell count, a condition known as aleukemic leukemia [2].

We report a case of B-cell acute lymphoblastic leukemia (ALL) with pancytopenia and lactic acidosis that responded only to chemotherapy. Patient also had associated RTA due to leukemic infiltrates in the kidneys.

## 2. Case Report

62-year-old female with past medical history of anemia presented with complaint of weakness and dizziness that started a week prior to admission, associated with > 20 lbs. of weight loss over 1 year. Upon admission, no specific clinical findings were noted except for reddish annular spots on the right lower extremities. Blood pressure was 169 / 72; pulse was 102 bpm; respiratory rate was 18 breaths/ minute; temp was 98.3 F; pulse ox was 100% on R/A.

Initial laboratory data revealed the data in Table 1.

Based on the results in Table 1, the serum anion gap is 21.5. However, the delta/delta ratio is ~0.74 which indicates that the patient has mixed anion gap and non-anion gap metabolic acidosis. The positive urine anion gap (36) and urine PH > 6 in the presence of metabolic acidosis suggest a renal involvement represented as RTA. Furthermore, we calculate the urine osmolar gap (UOG) using the following formula:

UOG = measured urine osmolality - ((2 *∗* (urine Na + urine K)) + (urine urea nitrogen / 2.8) + (urine glucose / 18)) which would create a urine osmolar gap of 95.43 mOsm/kg which further suggests the distal RTA.

Additionally, the patient had a bone marrow biopsy which showed markedly hypercellular bone marrow with 70% B-lymphoblast which is consistent with B-ALL. Staining is positive for TdT, PAX5, CD79a, and CD10. Cytological studies could not be performed due to dry tap.

Peripheral blood smear showed only few target cells.

**Figure 1:**
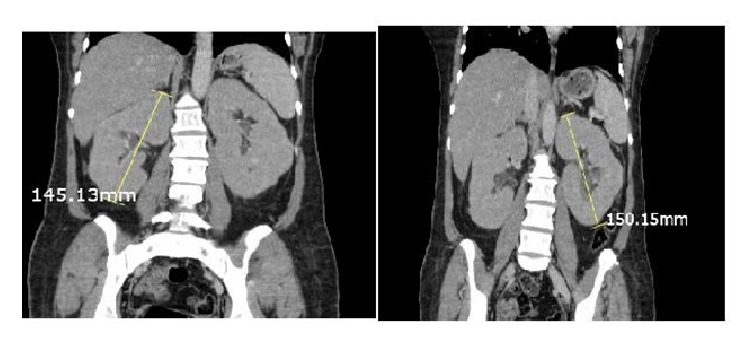
The enlargement of the kidneys bilaterally prior to chemotherapy.

Initial CT scan of abdomen was significant for enlargements of the kidneys bilaterally (see Figure 1).

Table 2 shows the hospital course for the management of lactic acidosis.

Based on Table 2, metabolic acidosis was first managed with fluid replacement and sodium bicarbonate while searching for possible causes of lactic acidosis. Lactic acidosis improved with fluids and bicarbonate replacement. However, the complete resolution was achieved only after chemotherapy with hyperfractionated chemotherapy of cyclophosphamide, vincristine, doxorubicin, and dexamethasone (hyper-CVAD) with intrathecal prophylaxis methotrexate was started. Patient received a total of 8 cycles of hyper-CVAD chemotherapy.

She had her bone marrow biopsy done after 6 cycles and was found to be in complete remission.

## 3. Discussion

Lactic acidosis results from an imbalance between lactic production and utilization. Lactic acid usually forms under anaerobic condition that shifts the pyruvate in the direction of lactate via lactate dehydrogenase. The most common causes of anaerobic metabolism are hypovolemia, hypoxia, cardiac failure, and sepsis [3]. In our case patient has saturation of 100% on RA, with normal vital signs except for mile elevation in blood pressure, septic work-up was negative, and echo showed normal Ejection Fraction: 55-60%. However, lactic acidosis did not respond to IV fluid replacement.

**Figure 2:**
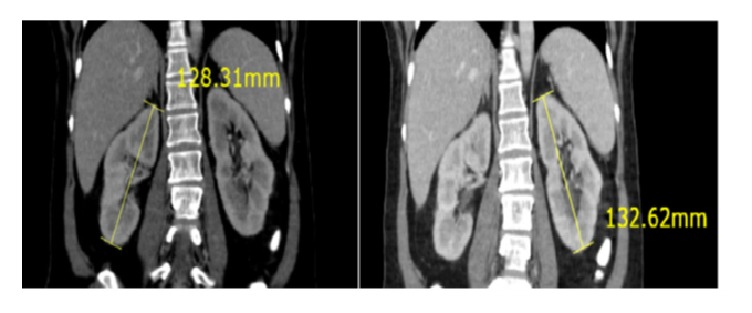
The change in size of the kidneys bilaterally after chemotherapy.

After lactic acid is produced, it is utilized mainly by the liver and by the kidneys to a lesser extent which makes metastasis to the liver or kidneys a potential cause for lactic acidosis in malignancies. Literature review revealed that only 20 cases of leukemia associated with lactic acidosis had liver involvement. And, 2 cases reported kidney involvement, whereas only 2 cases had both liver and kidney involvement [4, 5]. In our reported case initial imaging showed enlarged fatty liver and revealed enlargement of both kidneys. Repeated CT scan after 6 cycles of hyper-CVAD showed that kidney size has decreased with almost 2 cm difference [see Figures 1 and 2]. Kidney involvement in our reported case was responsible for the non-anion gap part of metabolic acidosis which mandates further search for the cause of acidosis.

Lactic acidosis in malignancies can also result from underperfusion of wide burden tumor or increased rate of aerobic glycolysis by neoplastic cells (Warburg effect). Burden of tumor is better assessed in solid tumors, but in hematologic malignancies cell count can be considered the best alternative. Out of the 26 reported cases cell count was either normal or elevated in 18 of them [4, 5]. In our case initial work-up included complete blood count which revealed pancytopenia.

**Figure 3:**
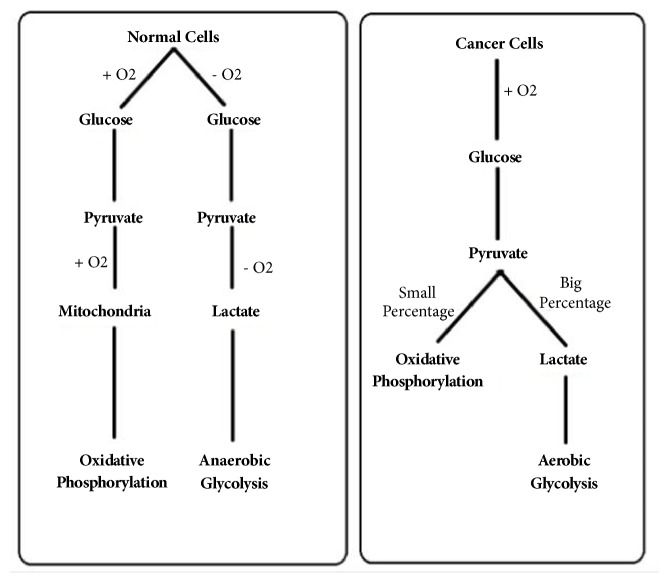
Comparison in the metabolism pathway between normal cells and neoplastic cells.

Warburg effect is a phenomenon that describes the unique metabolism in malignant cells. Malignant cells prefer to metabolize pyruvate into lactic acid direction even in the presence of oxygen, a process known as aerobic glycolysis [see Figure 3]. The primary goal of the process is not generating energy (ATP) but rather using products of aerobic glycolysis as building blocks to produce new daughter cell, whereas in the presence of oxygen nonproliferating cells tend to metabolize glucose through mitochondrial tricarboxylic acid (TCA) cycle followed by series of electron transport chain reactions known as oxidative phosphorylation with the primary goal being to maximize ATP production formed out of each molecule of glucose [6].

Many theories were proposed to explain this effect. Warburg who first described this effect in the early 1920s hypothesized that since cancer cells tend to be dysplastic, this effect results from mitochondrial dysfunction which subsequently impairs the processes (TCA /ETC) that take place in the micro-organelle. Therefore, the metabolism of glucose will shift towards fermentation of glucose into lactate. However, subsequent research showed that the mitochondria and their function are intact in most cancer cells [7].

Further research was able to recognize mutations involved in glucose metabolism inside cancer cells. These include the PI3K signaling pathway [7] and overexpression of Hexokinase-2 (HK2) [8], whereas another involved pathway is pyruvate kinase (PK)–M2 that represents the embryonic isoform of PK [9, 10]. Absence of these mutations in normal cells sheds light on the factors that may play an important role in establishing Warburg effect in proliferating cells.

With future research, different theories might be proposed in order to explain this effect. We hypothesize that the mutations responsible for Warburg effect result in mediators that alternate the metabolic pathway in the cancer cells. Being aware of these mediators can be a future promise for the new era of chemotherapy.

## 4. Conclusion

Lactic acidosis is a metabolic disorder that has different etiologies. It has been reported with malignancies including leukemia with high cell count. However, our case has some unique features including having AG and NAGA simultaneously, RTA due to leukemia infiltration of the kidneys, AG resulting from the unique metabolism of malignant cells, and resolution of both types of acidosis only after starting chemotherapy. Warburg effect is a big contributor to lactic acidosis in malignancy. Our case illustrates that this effect can be seen even with aleukemic Leukemia and suggests tumor load may not be needed for this phenomenon to occur.

## Figures and Tables

**Table 1:** Initial blood work results.

**Name of test**	**Reading**	**Reference range**
VBG PH	7.24	7.36 – 7.44
VBG PCO2	26 mmhg	36 – 44
VBG HCO3	11.1 mmol/L	22 – 66
VBG Base excess	-15.5 mmol/L	-2 - 3
Lactic acid	12.3 mmol/L	0.5 - 2.2

WBC	2.3 K/mm3	4.5 – 11
HGB	6.4 g/dl	12 – 16
HCT	17.3 %	36 – 42
PLTs	77 K/mm3	140 – 440
MCV	124.4 U3	80 – 100
RDW	16.2 %	0.5 - 16.5
Segs	33 %	36 – 75
Lymphs	62 %	24 – 44
Atypical Lymphs	1 %	0 – 7
Monocytes	2 %	4 – 10
Eosinophil	1 %	0 – 5
Basophil	1 %	0 – 2
Retic count	4.9 %	0.5 – 2
PT	13.8 sec	12.2 – 14.9
INR	1.1	1
PTT	28.2 sec	21.3 - 35.1

Na+	141 Meq/L	135 – 145
K+	3.7 Meq/L	3.5 – 5
Chloride	109 Meq/L	98 – 107
CO2	11 Meq/L	21 – 31
Blood glucose	101 mg/dl	70 – 105
BUN	23 mg/dl	7 – 23
Creatinine	1.18 mg /dl	0.60 – 1.30
Calcium	8.8 mg/dl	8.6 – 10.3
Total protein	6 g/dl	6.4 – 8.4
Albumin	3.8 g/dl	3.5 – 5.7
ALP	69 IU/L	34 – 104
AST	24 U/L	13 – 39
ALT	31 U/L	7 – 25
LDH	185 U/L	140 – 271
Serum osmolarity	297 mOsm/ Kg	283 – 299

Urine Na+	81 Meq/L	15 – 237
Urine K+	21 Meq/L	22 – 164
Urine CL-	24 mmol/L	24 – 255
Urine PH	6.5	5-8
Urine Osmolality	628 mOsm/kg	50 – 900
Urine glucose	Neg (mg/dl)	Negative

**Table 2:** Explanation of the hospital course management of lactic acidosis.

Date	Management of lactic acidosis	Lactate acid level MMOL/L	CO2 level MEQ/L
1^st^ day	0.9 % Normal saline	12.3	11

2^nd^ day	Normosol-R *∗*	11	11

1^st^ week	Dextrose 5% + sodium bicarbonate IV		17

2^nd^ week	0.9 % Normal saline + Sodium Bicarbonate and 1st cycle of chemotherapy(Hyper-CVAD )*∗∗*, with Intrathecal Methotrexate		13

3^rd^ week	Few days after 1^st^ cycle of Hyper CVAD with intrathecal Methotrexate	6.3	25

5^th^ week	0.45 normal saline + Sodium bicarbonate+ 2nd cycle of Hyper CVAD	-* *-	28

8^th^ week	4th cycle of Hyper CVAD		24

Discharge	-* *-	-* *-	25

*∗*Each 100 mL of Normosol-R contains sodium chloride, 526 mg; sodium acetate, 222 mg; sodium gluconate, 502 mg; potassium chloride, 37 mg; and magnesium chloride hexahydrate, 30 mg. **∗∗**Hyper-CVAD: hyper-fractionated chemotherapy of cyclophosphamide, vincristine, doxorubicin, and dexamethasone.
